# Glutamate Uptake Triggers Transporter-Mediated GABA Release from Astrocytes

**DOI:** 10.1371/journal.pone.0007153

**Published:** 2009-09-24

**Authors:** László Héja, Péter Barabás, Gabriella Nyitrai, Katalin A. Kékesi, Bálint Lasztóczi, Orsolya Tőke, Gábor Tárkányi, Karsten Madsen, Arne Schousboe, Árpád Dobolyi, Miklós Palkovits, Julianna Kardos

**Affiliations:** 1 Department of Neurochemistry, Institute of Biomolecular Chemistry, Chemical Research Center, Hungarian Academy of Sciences, Budapest, Hungary; 2 Laboratory of Proteomics, Institute of Biology, Eötvös Loránd University, Budapest, Hungary; 3 Department of Physiology and Neurobiology, Eötvös Loránd University, Budapest, Hungary; 4 Department of Molecular Spectroscopy, Institute of Structural Chemistry, Chemical Research Center, Hungarian Academy of Sciences, Budapest, Hungary; 5 Department of Pharmacology and Pharmacotherapy, Faculty of Pharmaceutical Sciences, University of Copenhagen, Copenhagen, Denmark; 6 Laboratory of Neuromorphology and Neuroendocrinology, Semmelweis University and Hungarian Academy of Sciences, Budapest, Hungary; University of North Dakota, United States of America

## Abstract

**Background:**

Glutamate (Glu) and γ-aminobutyric acid (GABA) transporters play important roles in regulating neuronal activity. Glu is removed from the extracellular space dominantly by glial transporters. In contrast, GABA is mainly taken up by neurons. However, the glial GABA transporter subtypes share their localization with the Glu transporters and their expression is confined to the same subpopulation of astrocytes, raising the possibility of cooperation between Glu and GABA transport processes.

**Methodology/Principal Findings:**

Here we used diverse biological models both *in vitro* and *in vivo* to explore the interplay between these processes. We found that removal of Glu by astrocytic transporters triggers an elevation in the extracellular level of GABA. This coupling between excitatory and inhibitory signaling was found to be independent of Glu receptor-mediated depolarization, external presence of Ca^2+^ and glutamate decarboxylase activity. It was abolished in the presence of non-transportable blockers of glial Glu or GABA transporters, suggesting that the concerted action of these transporters underlies the process.

**Conclusions/Significance:**

Our results suggest that activation of Glu transporters results in GABA release through reversal of glial GABA transporters. This transporter-mediated interplay represents a direct link between inhibitory and excitatory neurotransmission and may function as a negative feedback combating intense excitation in pathological conditions such as epilepsy or ischemia.

## Introduction

Maintenance of the balance between γ-aminobutyric acid (GABA) mediated inhibition and l-glutamate (Glu) mediated excitation is of crucial importance under normal and pathological conditions in the brain. Although operationally independent, the biochemically integrated GABA*ergic* and glutamat*ergic* neurotransmitter systems do interplay at cellular and sub-cellular levels [1]–[6]. The steady control over the extracellular concentrations of Glu and GABA is crucial for cell viability. This task is performed by Glu and GABA transporters that remove the neurotransmitters from the extracellular space utilizing the downhill transport of Na^+^. Glu transporters (EAATs) are predominantly localized to astrocytes [7] near the synaptic cleft [8]. Therefore proper function of EAATs is essential and represents a critical component in the neuroprotective role that astrocytes offer to neurons [9]. In contrast to Glu, GABA is predominantly taken up by neurons through the GABA transporter subtype 1 (GAT-1). Due to the prevalence of neuronal GABA uptake, GAT-1 used to be in the focus of transporter research for decades. As a consequence, little is known about the role of GAT subtypes localized to glial cells (GAT-2, GAT-3) despite their capability to markedly influence neuronal excitability [10] and the therapeutic potential of GAT-3 up-regulation in epilepsy [11], [12].

In the present study, we explore the transport properties of glial Glu and GABA transporter subtypes and the role they might play in establishing the crosstalk between glutamat*ergic* and GABA*ergic* neurotransmissions. Applying diverse biological models at different levels of complexity in combination with different analytical, pharmacological and anatomical approaches, we demonstrate the existence of a previously unrecognized mechanism through which astrocytes exchange extracellular Glu for GABA by a concerted action of glial Glu and GABA transporters.

## Results

### Interplay between glial Glu and GABA transport processes *in vitro*

**Figure 1:**
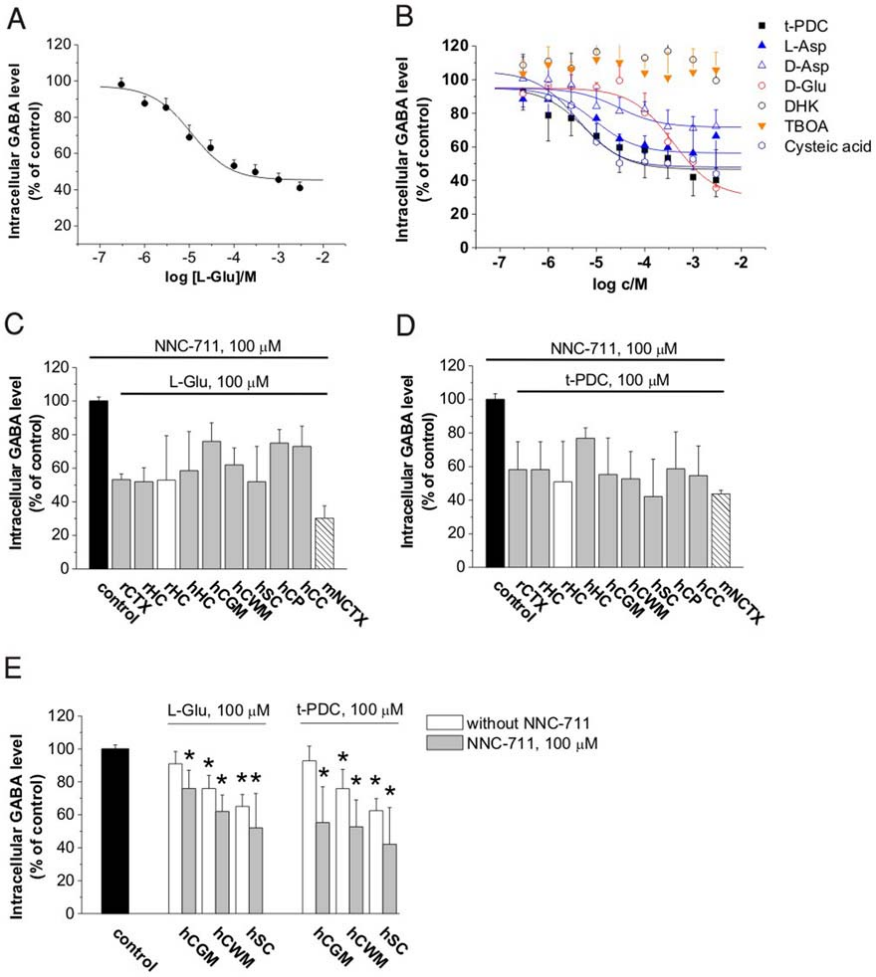
Cytosolic GABA level as determined by [^3^H]GABA uptake in the presence of 100 µM NNC-711. (A) Inhibition of [^3^H]GABA uptake following 10 min preincubation with Glu in rat cerebrocortical NPMV fraction (n = 7). IC_50_ value for Glu: 11.0±0.1 µM. (B) Inhibition of [^3^H]GABA uptake following 10 min preincubation with different EAAT substrates and inhibitors in rat cerebrocortical NPMV fraction (n = 3–6). (C and D) Decreased intracellular GABA level following Glu (C) or t-PDC (D) application in rat and human NPMVs (gray), from acute rat hippocampal slice (empty bar, n = 7) and from neuronal culture from mouse neocortex (striped bar, n = 4). Abbreviations used for brain regions: rat cerebrocortical neocortex (rNCTX, n = 6), rat hippocampus (rHC, n = 3), human hippocampus (hHC, n = 6), human cortical gray matter (hCGM, n = 6), human cortical white matter (hCWM, n = 6), human spinal cord white matter (hSC, n = 6), human choroid plexus (hCP, n = 6), human corpus callosum (hCC, n = 6). All drug applications significantly differ from the control (P<0.01). (E) Intracellular [^3^H]GABA level in the absence and presence of 100 µM NNC-711 during application of Glu (100 µM) or t-PDC (100 µM) in NPMV fractions from human cortical gray matter (hCGM, n = 6), human cortical white matter (hCWM, n = 6) and human spinal cord white matter (hSC, n = 6). Asterisks: P<0.05.

We explored the potential interplay between glial Glu and GABA transport processes in the presence of a specific [13], [14], [15], non-transportable blocker of GAT-1, 1,2,5,6-tetrahydro-1-[2-[[(diphenylmethylene)amino]oxy]ethyl]-3-pyridinecarboxylic acid (NNC-711) to pharmacologically isolate GAT-2/3 transporter function. Strikingly, selective blockade of the dominant GABA transporter subtype, GAT-1 by NNC-711 revealed the existence of a Glu-induced inhibition of [^3^H]GABA uptake into native plasma membrane vesicles (NPMV), isolated from rat cerebral cortex. Preincubation of vesicles with Glu for 10 minutes resulted in 52% inhibition of GABA uptake (Figure 1A). Besides Glu, other EAAT substrates (t-PDC, l-Asp, d-Asp, d-Glu, cysteic acid), but not the non-transportable inhibitors dihydrokainic acid (DHK) or dl-*threo*-β-benzyloxyaspartic acid (TBOA) were also able to affect GABA transport in the rat cortical NPMV (Figure 1B). Furthermore, amino acids (l-Leu, d-Leu, l-Ser, d-Ser), substrates of GABA transporter homologues (serotonin, dopamine), an intermediate of the GABA shunt (succinate) and an EAAT2 inducer β-lactam antibiotics (penicillin) did not affect GABA transport (IC_50_>1 mM). It is noteworthy that we did not observe an altered Glu uptake following GABA application either in control conditions or in the presence of NNC-711 and/or TBOA in rat cerebrocortical NPMV fractions (data not shown).

To explore the presence of EAAT mediated [^3^H]GABA uptake inhibition, we studied NPMVs from different brain areas. In human brain tissue samples both Glu (Figure 1C) and t-PDC (Figure 1D) were able to inhibit [^3^H]GABA uptake in all investigated areas including hippocampus, cortex, cortical gray and white matter, spinal cord white matter, choroid plexus and corpus callosum in different mammalian species (rat, mouse and human) as well as at different levels of complexity such as in brain tissue homogenates (NPMV), in isolated slices and in primary neuronal cultures that do express EAAT1/2 [16]. Notably, in brain regions with high glial content (cortical white matter, spinal cord white matter) Glu and t-PDC did inhibit [^3^H]GABA uptake even in the absence of NNC-711 (Figure 1E). Most of these experiments aimed to characterize the molecular parameters of the interplay between Glu and GABA transport processes were performed on NPMV fractions. However, the results are expected to be valid in isolated brain tissue slices as well because both Glu and t-PDC were found to be equally potent inhibitors of GABA uptake in rat hippocampal NPMVs and in freshly isolated (acute) rat hippocampal slices (Figure 1C, D). These results clearly demonstrate the existence of a Glu-induced GABA transport process throughout the brain.

### Interplay between glial Glu and GABA transport processes *in vivo*

**Figure 2:**
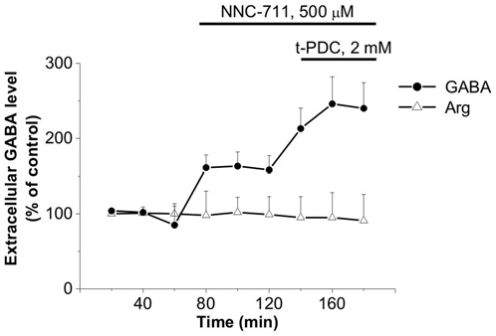
Elevation of [GABA]_o_ in the rat hippocampus *in vivo* following NNC-711 and t-PDC administration (n = 10, P = 0.019 (t-PDC vs. NNC-711), NNC-711: 160±18, t-PDC : 233±33, % of control). [Arginine]_o_ was used as a control for possible non-specific release (n = 10, P = 0.6, NNC-711: 100±25, t-PDC: 94±32, % of control).

We tested whether the activation of EAATs alters GABA transport *in vivo*. According to the *in vitro* results, application of the Glu transporter substrate t-PDC resulted in an increased extracellular GABA level ([GABA]_o_) in the rat hippocampus *in vivo* (Figure 2). The substantial increase of the tightly controlled [GABA]_o_ [17] following t-PDC administration was comparable to that evoked by GAT-1 blockade (Figure 2), predicting a significant consequence of the interplay between the Glu and GABA transport processes. To demonstrate that increase in extracellular GABA level is due to specific t-PDC effect, we measured the level of arginine as a reference amino acid. Arginine level did not change significantly during either NNC-711 or t-PDC application. It is worth noting that the extracellular concentration of applied drugs is lower than the concentration set in the microdialysis probe. Based on substance recovery curves [18], we estimate the extracellular concentration of NNC-711 and t-PDC to be 100 µM and 400 µM, respectively. Therefore the presence of the Glu-dependent GABA transport process is not restricted to *in vitro* model systems, it is present in the *in vivo* functional brain.

### Glu transporter activation induces GABA release

**Figure 3:**
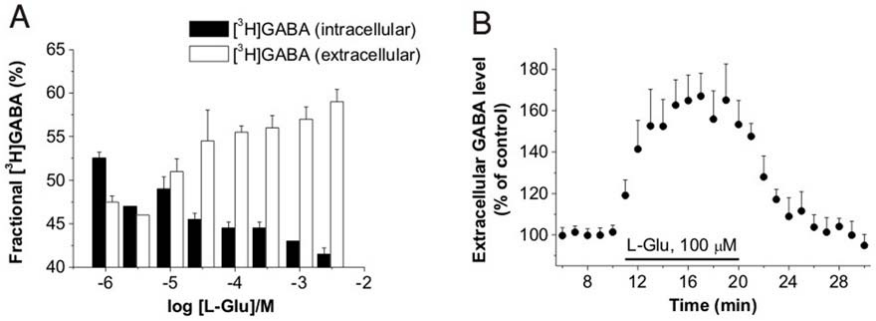
Glu induces GABA release (A) Glu uptake decreases cytosolic GABA level as determined by [^3^H]GABA release from NPMV vesicles preloaded with [^3^H]GABA (n = 3). IC_50_ value for Glu: 13.1±1.6 µM. (B) Elevation of [GABA]_o_ in acute rat hippocampal slices during application of 100 µM Glu (n = 6; 149±14% of pre-stimulus control).

In all the above experiments, intra- or extracellular GABA content was determined. Apparent inhibition of GABA uptake can be the result of either inhibited uptake or enhanced release. To decide between these possibilities, two different experimental approaches were applied. In the steady-state experiment, rat cortex NPMVs were preloaded with [^3^H]GABA and extra- and intracellular [^3^H]GABA contents were determined after 10 min incubation with different concentrations of [^14^C]Glu. Glu application dose-dependently triggered the release of the preloaded GABA (Figure 3A). In the superfusion experiment, acute rat hippocampal slices were preloaded with [^3^H]GABA and the extracellular GABA content was recorded in a microvolume superfusion system. The extracellular [^3^H]GABA level elevated to an average 149±14% of pre-stimulus control following application of 100 µM Glu (Figure 3B). It is noteworthy that although this Glu concentration is much higher than the steady state extracellular [Glu], it reflects the concentration sensed by EAATs. After synaptic release, the concentration of Glu in the synaptic cleft may elevate to 1–5 mM [19]. Since EAATs are located near the release site^8^, they are exposed to relatively high Glu concentrations (160–190 µM) [20]. In both experiments, Glu application directly evoked the release of the preloaded labeled GABA, demonstrating that the Glu-dependent GABA transport is a GABA release process.

### Glu-induced GABA release cannot be blocked by Glu receptor antagonists

**Figure 4:**
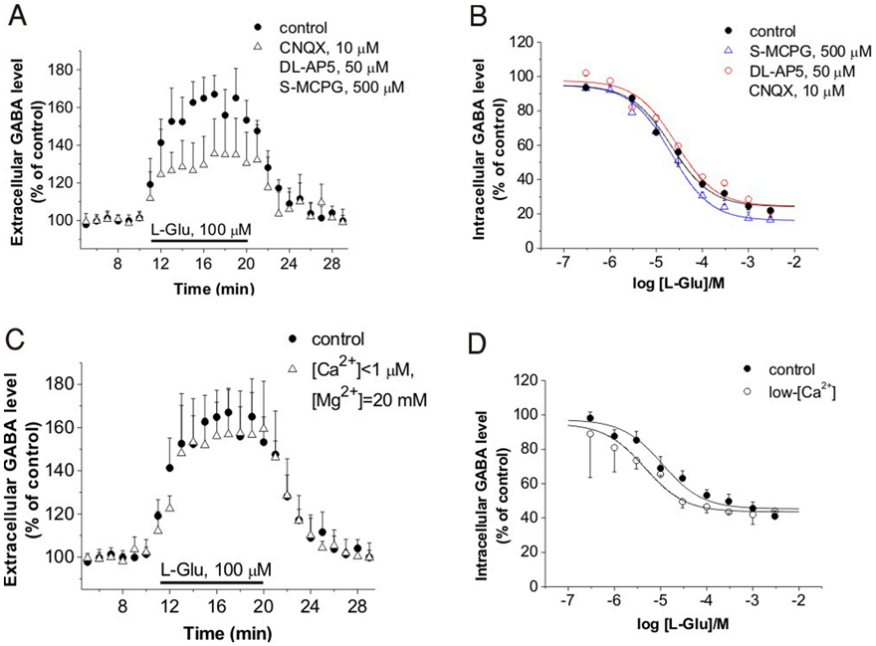
Glu-induced GABA release is not mediated by vesicular release. (A) Elevation of [GABA]_o_ in acute rat hippocampal slices in control conditions (n = 6) and in the presence of glutamate receptor antagonists (n = 7; P = 0.014; 128.5±13.4% of pre-stimulus control). (B) Cytosolic GABA level as determined by [^3^H]GABA uptake in the presence of 100 µM NNC-711 and Glu receptor antagonists in rat cerebrocortical NPMV fractions (n = 4; P = 0.82) (C) Elevation of [GABA]_o_ in acute rat hippocampal slices in control conditions (n = 6) and in low-[Ca^2+^]/high-[Mg^2+^] buffer (n = 5; P = 0.91; 148.3±15.5% of pre-stimulus control). (D) Cytosolic GABA level as determined by [^3^H]GABA uptake in the presence of 100 µM NNC-711 in low-[Ca^2+^] buffer in rat cerebrocortical NPMV fractions (n = 3; P = 0.49). Low-[Ca^2+^] buffer contained 145 mM NaCl, 5 mM KCl, 20 mM MgCl_2_, 10 mM glucose and 20 mM HEPES (pH 7.5).

To investigate the possibility whether GABA release is due to glutamat*ergic* stimulation of inhibitory neurons, Glu receptor antagonists were applied. NMDA (dl-2-amino-5-phosphonopentanoic acid: dl-AP5, 50 µM), AMPA/kainate (6-cyano-7-nitroquinoxaline-2,3-dione: CNQX, 10 µM) and metabotropic ((*S*)-α-methyl-4-carboxyphenylglycine: (*S*)-MCPG, 500 µM) Glu receptor antagonists partially inhibited the Glu-induced GABA release (Figure 4A). However, 58% of average [GABA]_o_ increase remained in the presence of Glu receptor antagonists.

In the rat cortical NPMV fraction, application of Glu receptor antagonists did not alter the effect of Glu on GABA transport (Figure 4B). In addition, blockade of the chloride influx through GABA_A_ receptors by the antagonist (1*S*,9*R*)-bicuculline methiodide and GABA_B_ receptor-mediated inhibition of GABA release by the agonist (*R*)-baclofen did not modulate the effect of Glu on the cytosolic GABA level (n = 4; P = 0.86; data not shown).

These data conclusively suggest that Glu-induced GABA release is not triggered by Glu receptor mediated depolarization.

### Glu-induced GABA release is independent of extracellular Ca^2+^

Major mechanisms for neurotransmitter release are Ca^2+^-dependent vesicular release and reversal of plasma membrane transporters [21]. To further confirm that Glu-induced GABA release is not due to glutamat*ergic* stimulation of inhibitory neurons, we examined GABA efflux from rat hippocampal slices in high-[Mg^2+^]/low-[Ca^2+^] media (Figure 4C) and in the presence of 50 µM Cd^2+^ salt in the superfusion (n = 3; P = 0.82; 143±10% of pre-stimulus control, data not shown). We also determined GABA uptake in rat cortical NPMV under low-[Ca^2+^] conditions (Figure 4D). All these experimental approaches indicated that the mechanism responsible for Glu-induced GABA release is Ca^2+^-independent, therefore it is not a vesicular release.

### Glu-induced GABA release is independent of Glu decarboxylase

**Figure 5:**
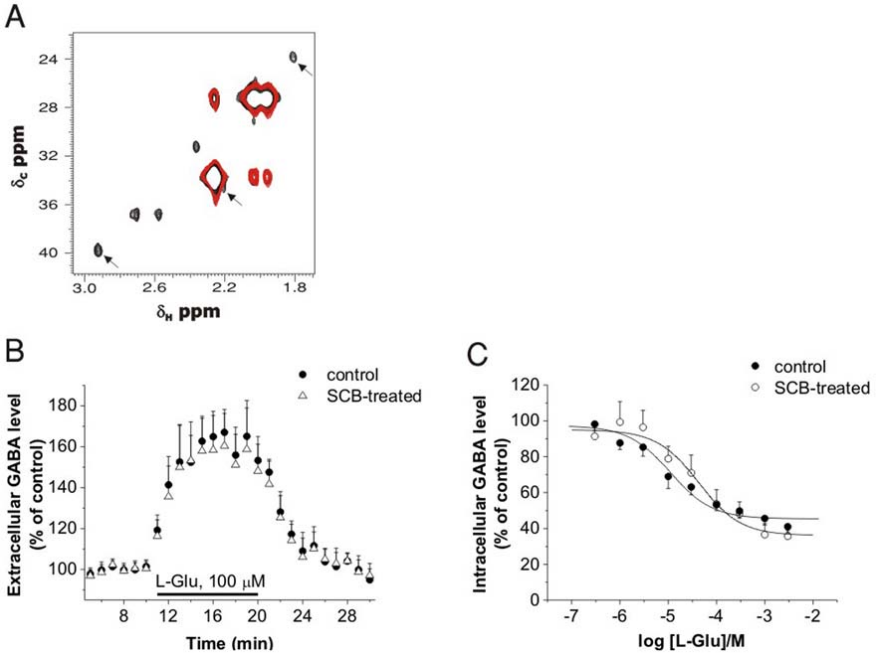
Glu-evoked GABA release is independent of glutamate decarboxylase. (A) Contour plot representations of gradient enhanced ^1^H/^13^C heteronuclear single quantum coherence (^13^C-HSQC) spectra of extracellular fluid sample from rat cerebrocortical NPMV after incubation with [U-^13^C/^15^N]Glu for 30 min in control (black) and semicarbazide (SCB)-treated animal (red). GABA resonances (arrows) do not appear in the samples from SCB-treated animal. (B) Elevation of [GABA]_o_ in acute rat hippocampal slices in control (n = 6) and SCB-treated animals (n = 7; P = 0.54; 145±14% of pre-stimulus control). (C) Intracellular [^3^H]GABA level as determined by [^3^H]GABA uptake in the presence of 100 µM NNC-711 in control condition and in SCB-treated animals (n = 3, P = 0.85).

Previous studies reported a receptor-independent, metabolic pathway through which extracellular Glu may elevate [GABA]_o_ [4], [5], [22]. This mechanism is thought to involve neuronal uptake of Glu through EAAT3, metabolic conversion to GABA by Glu decarboxylase (GAD) and release of this newly synthesized GABA. To decide whether this mechanism contributes to the Glu-induced GABA release, the source of GABA in rat cortical NPMV fraction was determined by two dimensional NMR methods following [^13^C]Glu administration. It was found that ∼5% of extracellularly applied [^13^C]Glu was converted to [^13^C]GABA and released into the extracellular space (Figure 5A). Although in situ formation of GABA elucidates Glu-induced GABA release, it is highly unlikely that other EAAT substrates like t-PDC and cysteic acid can replace Glu in metabolic pathways. Therefore, an additional mechanism should take place in action. Indeed, the [^13^C]GABA formation was completely blocked by treatment with the GAD inhibitor semicarbazide (SCB) in rat cortical NPMV fraction (Figure 5A). In acute hippocampal slices SCB treatment also significantly reduced [^13^C]GABA formation from [^13^C]Glu (data not shown). However, SCB treatment did not affect [^3^H]GABA release either from acute hippocampal slices (Figure 5B) or from rat cortical NPMV fractions (Figure 5C). Since different pools of GABA (newly synthesized vs. previously captured, respectively) were labeled in NMR and radiotracer measurements, the relative weights of the GAD-mediated and the GAD-independent processes were obtained by measuring the total pool of GABA using HPLC studies from rat cerebrocortical NPMV samples. We found that application of 300 µM Glu increased the extracellular GABA level by 46±3% in control (n = 3, P = 0.003) and by 35±6% in SCB-treated rats (n = 3, P<.001). The moderate decrease of [GABA]_o_ elevation in SCB-treated rats (35% vs. 46% in control animal) suggests that the GAD-independent mechanism represents the major route in Glu-induced GABA release.

### Localization of GAT-1 and GAT-3 in the hippocampus

**Figure 6:**
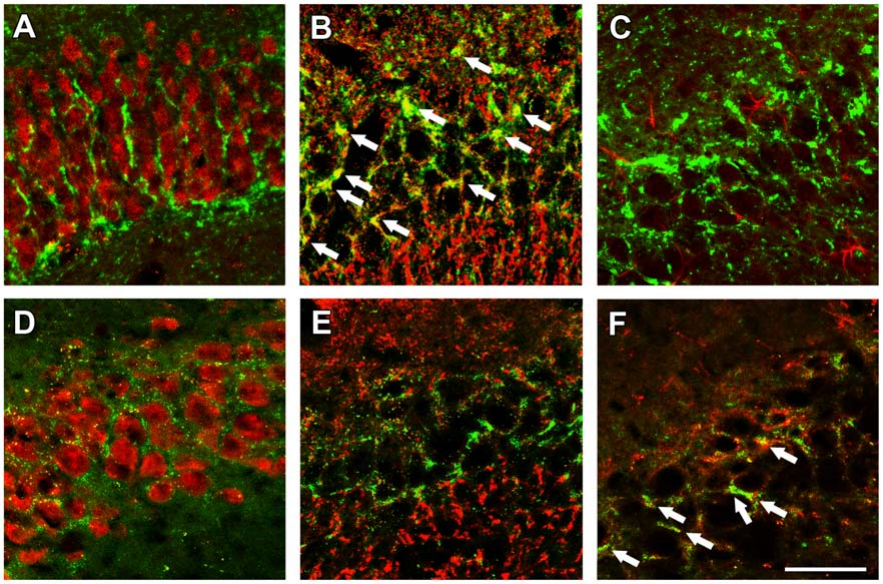
GABA transporter subtypes double labeled with neuronal and glial markers in hippocampal sections. GAT-1 (A–C) and GAT-3 (D–F) are labeled with green while NeuN (A, D), synaptophysin (B, E), and GFAP (C, F) are red. The yellow labeling in B and F demonstrate co-localization of GAT-1 with synaptophysin and GAT-3 with GFAP (some colocalization sites are marked by arrows). Scale bar  = 50 µm.

To explore the specific localization of different GABA transporter subtypes possibly involved in the Glu-induced GABA release, we investigated the expression of GAT-1 and GAT-3 by immunostaining (Figure 6). GAT-1 immunoreactivity was localized throughout the hippocampus in puncta while in the pyramidal cell layer labeled fibers can be followed radially along the neuronal cell bodies. GAT-1 immunoreactivity was co-localized with synaptophysin (Figure 6B) suggesting its presynaptic localization. A much lower (∼10% of GAT-1) density of GAT-3 immunolabeling is present in the hippocampus. GAT-3 immunolabeling has only a small degree of co-localization with synaptophysin (Figure 6E) but shows a significant co-localization with glial fibrillary acidic protein (GFAP) suggesting its presence in astrocytes (Figure 6F).

### Glu-induced GABA release is mediated by GAT-2/3

**Figure 7:**
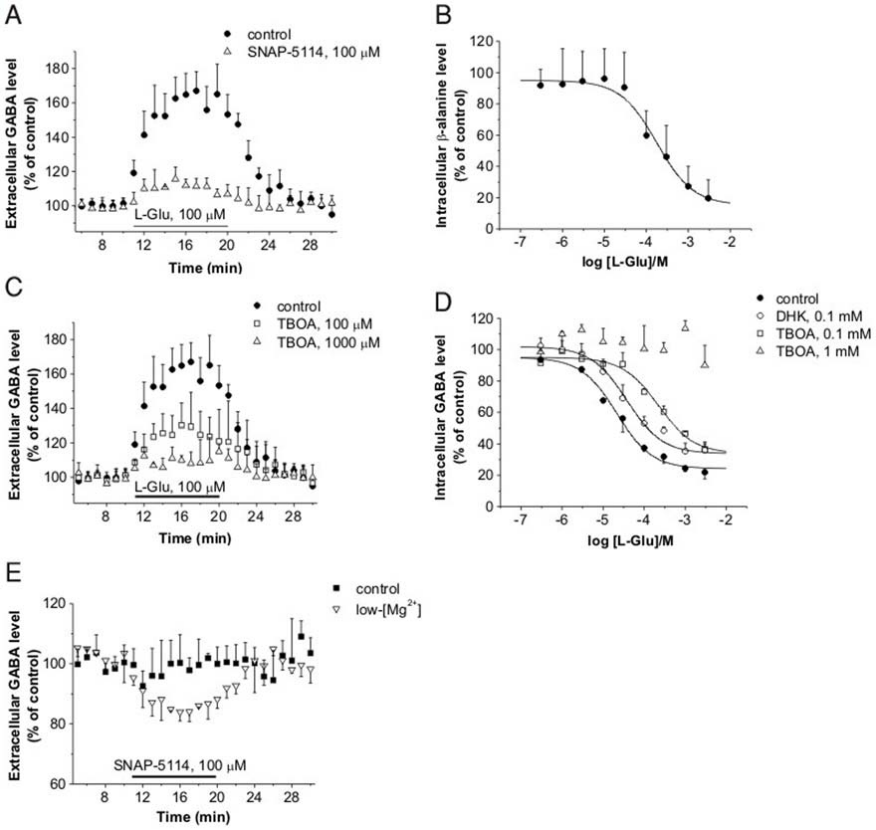
Glu-induced GABA release requires the concerted action of Glu and GABA transporters. (A) Elevation of [GABA]_o_ in acute rat hippocampal slices in control conditions (n = 6) and in the presence of the specific GAT-2/3 blocker, SNAP-5114 (n = 4, P<0.001; 109±4% of pre-stimulus control). (B) Cytosolic β-alanine level as determined by [^3^H]β-alanine uptake in rat cerebrocortical NPMV fraction (n = 3). (C) Elevation of [GABA]_o_ in acute rat hippocampal slices in control conditions (n = 6) and in the presence of the Glu transporter blocker, TBOA (100 µM: n = 4, P<0.001; 123±10% of pre-stimulus control, 1000 µM: n = 4, P<0.001; 109±1% of pre-stimulus control). (D) Cytosolic GABA level as determined by [^3^H]GABA uptake in the presence of 100 µM NNC-711 and Glu transporter blockers in rat cerebrocortical NPMV fraction (n = 3; P<0.001 for both DHK and TBOA). (E) Alteration of [GABA]_o_ in acute rat hippocampal slices in normal ACSF (control, n = 5, 100±6% of pre-application control) and in nominally Mg^2+^-free ACSF (low-[Mg^2+^], n = 5, P<0.001; 90±4% of pre-application control) during blockade of GAT-2/3 by the specific, non-transportable inhibitor SNAP-5114.

Since it was demonstrated before that Glu-induced GABA release is not through vesicular release, it is plausible to assume that it is mediated by reversal of GABA transporter. This hypothesis was further supported by the pharmacological profile of GABA uptake in rat cerebrocortical NPMV in the presence of NNC-711 (Table 1). To confirm this assumption, GABA efflux from rat hippocampal slices was determined in the presence of the specific, non-transportable GAT-2/3 inhibitor 1-[2-[*tris*(4-methoxyphenyl)methoxy]ethyl]-(*S*)-3-piperidinecarboxylic acid (SNAP-5114). SNAP-5114 mostly inhibited Glu from evoking GABA release in rat hippocampal slices (Figure 7A). The ability of Glu to reduce cytosolic level of the specific GAT-2/3 substrate β-alanine (Figure 7B) further reinforced the involvement of GAT-2/3.

**Table 1:** GABA release following Glu application is probably mediated by GAT-2/3. Pharmacological profile of GABA transport in rat cerebrocortical NPMV in the presence of 100 µM NNC-711.

	IC_50_ (µM)				
	GAT-1^a^	BGT a	GAT-2 a	GAT-3 a	Rat cerebrocortical NPMV^b^
β-alanine	2920	1100	66	110	26±4
Taurine	>1000	>1000	1270	2860	400±7
Nipecotic acid	24	2350	113	159	28±0.5
Hypotaurine	1010	536	52	73	20±0.4
SKF89976A	0.64	7210	550	4390	372±9
Betaine	>1000	173	>1000	>1000	>1000

a Borden,L.A. GABA transporter heterogeneity: pharmacology and cellular localization (1996) Neurochem Int. 4: 335–356.

b present study.

### Glu-induced GABA release requires preceding Glu uptake

The inability of non-transportable EAAT inhibitors to affect GABA transport in the rat cortical NPMV (Figure 1B) suggested that the translocation process through Glu transporters is required to evoke the GABA release. To examine this possibility, the non-transportable EAAT inhibitors, DHK and TBOA were applied to exclude substrate transport. Inhibition of Glu transporters by 100 µM and 1000 µM TBOA resulted in a concentration dependent inhibition of Glu-induced GABA release in hippocampal slices (Figure 7C). Because EAAT blockade leads to extracellular Glu accumulation and therefore increased activation of Glu receptors, this finding further confirms that Glu-induced GABA release is not due to receptor-mediated activation of inhibitory neurons. The effect of Glu on GABA transport in rat cerebrocortical NPMV fraction was also significantly reduced in the presence of the selective EAAT2 blocker DHK (100 µM, Figure 7D) and further reduced in the presence of the non-selective EAAT1-3 blocker TBOA (Figure 7D), indicating that Glu uptake is a prerequisite to GABA release.

### GABA is released through GAT-2/3 during enhanced Glu transmission

The above experiments clearly showed that activation of Glu uptake by exogenously applied Glu fosters GABA release through glial GAT-2/3. Next we explored whether the concentration reached by endogenous Glu is able to activate the Glu-GABA exchange mechanism. To specifically study the GABA release through GAT-2/3 we applied 100 µM SNAP-5114 to block this release route in control conditions without exogenously added Glu. Blockade of GAT-2/3 did not decrease the [^3^H]GABA release from the labeled cytosolic pool (Figure 7E), indicating that GAT-2/3 mediated GABA release does not contribute to [GABA]_o_. However, increasing synaptic Glu release by applying nominally Mg^2+^-free medium [23], [24] revealed a significant contribution of the Glu-GABA exchange mechanism to the overall GABA release (Figure 7E). In nominally Mg^2+^-free ACSF, application of 100 µM SNAP-5114 reduced the [^3^H]GABA release by 10% on average compared to the pre-application period (Figure 7E), suggesting that the Glu-GABA exchange mechanism can emerge simultaneously with the network activity.

## Discussion

The principal finding of this study is that the uptake of Glu is coupled to the subsequent reversal of the glial GABA transporters bringing about an elevation in the level of extracellular GABA. We were thus able to show, for the first time, [GABA]_o_ signals resulting from glial Glu uptake. We demonstrated the presence of this Glu-GABA exchange mechanism *in vivo* in the rat hippocampus and *in vitro* by measurements of GABA uptake and release in rat brain slices, in cultured mouse neurons as well as in rat or human brain suspensions from different areas, particularly in cortical gray and white matter, spinal cord, corpus callosum, choroid plexus and the hippocampus. We found that all Glu transporter substrates examined, but not non-transportable inhibitors were able to evoke GABA release.

### GABA release is triggered by Glu transporter and not by Glu receptor activation

We presented several lines of evidence to support that Glu-induced GABA release does not involve Glu receptor-mediated depolarization. The fact that Glu-induced GABA release is partially inhibited in the presence of Glu receptor antagonists in the radiotracer release experiments apparently imply that the underlying mechanism does involve the activation of Glu receptors. However, we have demonstrated that GABA release was unaffected by applying low-[Ca^2+^] conditions or by the presence of Cd^2+^, indicating that GABA is released by reversal of GABA transporters and not by vesicular release. Our explanation for the partial inhibition of Glu-induced GABA release by Glu receptor antagonists is that Glu application both induces the GABA release through the mechanism described here and also keeps the neurons in an enhanced activation state. The enhanced activation leads to increased Glu release and subsequently increased activation of the EAATs. By adding Glu receptor antagonists, the neuronal activity was reduced resulting in a smaller effect of EAAT activation on GABA release. The lack of Glu receptor mediated component in the Glu-induced GABA release is further confirmed by the fact that GABA release can be excluded by EAAT blockade. In case of substantial Glu receptor contribution to the mechanism, EAAT blockade should not have been complete, because the increasing extracellular Glu level and subsequent intensification of Glu receptor currents should have overwhelmed the blockade of the EAATs. Therefore we propose that GABA release is prominently induced by EAAT activation.

The lack of Glu receptor contribution to the evoked GABA release may be due to the localization of GAT-2/3 transporters. In the hippocampus, their expression is confined to protoplasmic astrocytes [25]. This astrocyte subpopulation expresses Glu transporters, but does not express Glu receptors [26]. The lack of Glu receptor expression on GAT-2/3 expressing protoplasmic astrocytes does explain the lack of Glu receptor contribution to the glial GABA release.

### Proposed model of Glu-induced GABA release

**Figure 8:**
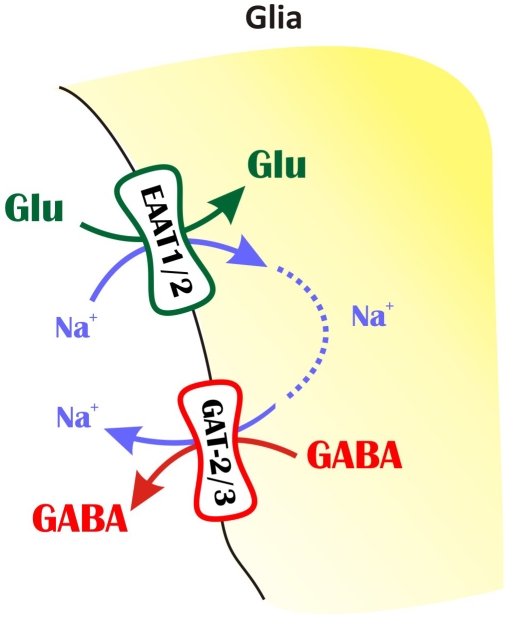
Schematic representation of the hypothesized Glu-GABA exchange mechanism. Glial uptake of Glu is coupled to the release of GABA. The mechanism supposes that the Na^+^ that is cotransported with Glu initiates GAT-2/3 transporter reversal.

To explain these findings, we propose a model for the mechanism underlying extracellular Glu-GABA exchange (Fig. 8). This model takes advantage of the phenomenon that GABA release can only be evoked by transportable EAAT substrates, recognizing the Glu translocation process itself as a prerequisite in inducing the release of GABA. Removal of Glu from the extracellular space is predominantly mediated by glial EAAT1 and EAAT2 and is coupled [27] to co-transport of 3 Na^+^/1 H^+^ and counter-transport of 1 K^+^, resulting in subsequent disruption of the resting electrochemical potential. Because GABA transport is also driven by Na^+^ gradient, the increased intracellular [Na^+^] may be capable of reversing GABA transporters. It is known from both theoretical [28] and experimental [29], [30] studies that GABA transporters operate close to their equilibrium potentials, therefore small perturbations in the extracellular or intracellular concentrations of GABA, Na^+^ or Cl^−^ may lead to reversal of GATs [30]. By the reversed action of glial GAT-2/3, intracellularly available GABA in astrocytes [31]–[34] can be released into the extracellular space. Since GAT-2/3 transporters are located outside the synapse [35], the released GABA is likely to activate the extrasynaptic GABA receptors located on dendrites [36] and may contribute to the tonic inhibition of neurons.

### Consequences of Glu-induced GABA release

The coupling between substrate activation of EAATs and subsequent GABA release implies that transportable and non-transportable inhibitors of Glu transport should have differential effects on neuronal viability. Indeed, this distinction was observed in several cases. In contrast to the non-transportable blocker DHK, the substrate t-PDC did not evoke neuronal damage *in vivo* even in very high concentration (25–100 mM), despite the fact that extracellular [Glu] elevation was similar [37] or even higher [38], [39] after t-PDC application than following DHK treatment. Also, *in vivo* administration of TBOA in the hippocampus was demonstrated to induce neuronal damage in the CA1 and the dentate gyrus, while t-PDC application did not produce cell death [40]. Beyond the primary benefit of turning excitation into inhibition, the Glu-GABA exchange also helps in the recovery of the transmembrane Na^+^ gradient without using ATP, thus profiting energy from neuroprotection. The maintenance of the Na^+^ homeostasis may in turn facilitate Glu uptake, allowing a substrate-induced increase of Glu transport activity without the need for protein synthesis [41].

### Physiological/pathophysiological role of Glu uptake-coupled GABA release

We have showed that GAT-2/3 mediated GABA release does not contribute to the [GABA]_o_ under normal, physiological conditions, but significantly emerges in low-[Mg^2+^] medium which is a standard *in vitro* model for epilepsy [24], [42], [43]. This would imply that the Glu-GABA exchange is primarily a pathophysiological mechanism. However, it is worth noting that network activity in *in vitro* brain slices is significantly reduced when compared to *in vivo* data [43], [44]. We measured average firing rate of hippocampal pyramidal cells of 0.6 Hz in normal ACSF and 3.5 Hz in low-[Mg^2+^] medium [43]. The latter is more comparable to in vivo firing rate of 3–5 Hz [44], suggesting that *in vivo* network activity may be sufficient to drive the Glu-GABA exchange. Nevertheless, the Glu-GABA exchange mechanism has the potential to limit Glu increase and/or counterbalance its effects under pathophysiological condition. The functional role of the Glu-GABA exchange may also be highlighted by the marked increase of GAT-3 expression in astrocytes, in the hippocampi of patients with temporal lobe epilepsy [25]. The dysfunction of the mechanism may also lie behind the impairment of the cross-talk between excitatory and inhibitory transport processes in temporal-lobe epilepsy [45], [46]. Treatments that target a mechanism which is up-regulated *in situ* in pathological conditions can represent an ideal strategy in drug development. As an example, the antiepileptic drugs clobazam and levetiracetam have been shown to up-regulate GAT-3 expression in the hippocampus [11], [12], highlighting the role of glia and glial transporters in maintaining the balance between inhibition and excitation [47]. We envision that the discovery of the glial Glu-GABA exchange will support the development of new pathomechanism-specific treatments for pathological conditions characterized by intense excitation, such as epilepsy or ischemia.

## Materials and Methods

Animals were kept and used in accordance with the European Council Directive of 24 November 1986 (86/609/EEC), the Hungarian Animal Act, 1998. All experiments involving animals were done by the approval of the Animal Testing Committee of the Chemical Research Center, Hungarian Academy of Sciences and by the approval of the Ministry of Agriculture and Rural Development, Hungary. All efforts were made to reduce animal suffering and the number of animals used.

The human brains were obtained from the Lenhossek Human Brain Program, Human Brain Tissue Bank, Budapest. Brains were taken from persons who had died without any known neurodegenerative diseases. The collection of brains and the microdissection of the brain samples for research have been performed by the approval of the Regional Committee of Science and Research Ethics of the Semmelweis University, Budapest (TUKEB:32/92) and the Ethics Committee of the Ministry of Health, Hungary, 2002 according to the principles expressed in the Declaration of Helsinki. Tissues were collected only after a family member gave informed (written) consent.

### Buffers

Buffers contained in mM; HEPES buffer: 145 NaCl, 5 KCl, 1 CaCl_2_, 1 MgCl_2_, 10 glucose and 20 HEPES (pH 7.5); ACSF: 129 NaCl, 3 KCl, 1.6 CaCl_2_, 1.8 MgSO_4_, 1.25 NaH_2_PO_4_, 21 NaHCO_3_, 10 glucose (pH 7.4); nominally Mg^2+^-free ACSF was prepared as control ACSF with no added Mg^2+^ (based on the Mg^2+^ contamination of the Ca^2+^ salts, we estimated the Mg^2+^ concentration of this buffer to be ∼1 µM).

### Slice preparation

Transverse, 400 µm thick hippocampal-entorhinal slices from 11–18 day old Wistar rats (Toxicoop, Budapest, Hungary) were prepared in modified artificial cerebrospinal fluid (75 mM sucrose, 87 mM NaCl, 2.5 mM KCl, 1.25 mM NaH_2_PO_4_, 7 mM MgSO_4_, 0.5 mM CaCl_2_, 25 mM NaHCO_3_, 25 mM glucose, continuously bubbled with 95% O_2_ +5% CO_2_ gas mixture) at 4°C, as described before [48]. Slices were incubated in an interface-type chamber that for 1 hour at 37°C (followed by incubation at room temperature) before performing the experiments. In experiments where [Mg^2+^] was lowered, slices were incubated in ACSF containing ∼100 µM Mg^2+^ and transferred to nominally Mg^2+^ free medium in the recording chamber 20–30 minutes before the start of the electrophysiological recording.

### [^3^H]GABA and [^3^H]β-alanine exchange in native plasma membrane vesicle (NPMV) fractions

Cerebral cortices of 4–6 weeks old male Wistar rats (Toxicoop, Hungary) and frozen human brain tissue samples (Hungarian Brain Tissue Bank, Budapest) were used to prepare NPMV fractions, that contain numerous membrane vesicles of different sizes and shapes, a few intact synaptosomes and free mitochondria [49]. Uptake studies were performed as follows: aliquots of NPMV fractions in HEPES buffer were preincubated with test compounds in the presence of NNC-711 (100 µM, Tocris) at 30°C for 10 min. Application of NNC-711 resulted in 86±3% reduction of the [^3^H]GABA uptake (n = 51). Afterward, preincubated solution was incubated with [^3^H]GABA (10 nM, Amersham) and GABA (90 nM, Sigma) for 1 min. In case of [^3^H]β-alanine uptake, incubation solution contained 100 nM [^3^H]β-alanine (Amersham) and 900 nM β-alanine (Sigma). After the incubation, samples were immediately filtered through glass fiber filters and washed twice with ice-cold HEPES buffer. The [^3^H]GABA level inside the plasma membrane vesicles was determined by liquid scintillation method. Although this method is generally used to measure GABA uptake, we applied it to follow GABA release due to the simplicity and reliability of the assay. We also determined GABA release by preloading [^3^H]GABA into NPMV vesicles and measuring the [^3^H]GABA level in the intracellular and extracellular milieu. The determined IC_50_ for Glu determined by the two assays did not differ significantly. Non-specific uptake was determined in the presence of 1 mM guvacine. The radioactivity of samples was counted in HiSafe 3 (Perkin Elmer) scintillation mixture.

### [^3^H]GABA exchange in neuronal cell cultures

Cortical neurons obtained from dissociated neocortex of 15 day old mouse embryos were cultured for 7 days as described by Hertz et al. [41]. Cytosine arabinoside (20 µM) was added to the cultures after 48 hours *in vitro* [50]. On the day of the experiment, the cell cultures were pre-incubated for 30 min with the test compound in physiologically balanced salt solution (137 mM NaCl, 2.7 mM KCl, 1 mM CaCl_2_, 1 mM MgCl_2_, 4.3 mM Na_2_HPO_4_, 1.4 mM KH_2_PO_4_, 6.5 mM glucose, pH 7.4) in the presence of 100 µM NNC-711, followed by the addition of [^3^H]GABA and GABA to a final concentration of 3 µCi/ml and 0.5 µM, respectively. After 3 min, the cells were washed, solubilized and used for measurements of radioactivity and protein contents [51].

### [^3^H]GABA exchange in rat hippocampal slices

Rat hippocampal slices were placed on the bottom of 6 ml sample holder tubes. 400 µl of solutions containing 10 nM [^3^H]GABA (Amersham) and 90 nM GABA (Sigma) were added onto the slices and incubated at 30°C for 1 min. Uptake was terminated by quenching with 4 ml ice-cold guvacine (1 mM, Sigma) solution and the slices were washed twice with ice-cold HEPES buffer. Afterwards, slices were vortexed in 400 µl HEPES buffer containing 5% Na-dodecylsulfate. Non-specific uptake was determined in the presence of 1 mM guvacine. The radioactivity of samples was counted in HiSafe 3 scintillation mixture.

### *In vivo* microdialysis and HPLC amino acid detection

Male Wistar rats (300–400 g) were anaesthetized with halothane (1% in air) and microdialysis probes were implanted into the ventral hippocampus (A: −5.0, L: 5.0, V: −8.0) as described previously [52]. To minimize tissue damage, the final position of the probes was reached slowly, in not less than 20 min. Perfusion of artificial cerebrospinal fluid, containing 144 mM NaCl, 3 mM KCl, 1 mM MgCl_2_, 2 mM CaCl_2_ (pH 7.4), was performed at a rate of 1 µl/min. The dialysate samples during 20 min were stored at −20°C. To establish the background amino acid concentration, each experiment started with the collection of three control samples with <10% variation. NNC-711 was applied bilaterally for 60 min followed by the simultaneous application of t-PDC and NNC-711 for 60 min. Concentration of amino acids was measured by pre-column derivatization of primary amino acids with orthophthalaldehyde (OPA, Sigma). Derivatization was performed at pH 10.4 in the presence of mercaptoethanol. OPA-derivatized amino acids were detected with fluorescence detector excitation wavelength 340 nm and emission wavelength 440 nm. Because of the instability of OPA derivatives, the HPLC technique was automated on an HP 1100 series system. Amino acids were separated on HP Hypersyl ODS reversed-phase columns (200×2.1 mm) filled with 5 µm C-18 spherical packing material. Eluent A was 0.1 M phosphate buffer containing 0.5% (vol/vol) tetrahydrofuran (Merck), pH 5.6, while eluent B contained 70% acetonitrile (Merck) in eluent A, pH 5.6. The gradient profile was: 12% B at 0 min, 23% B at 9 min, 36% B at 18 min, 100% B at 20 min, 100% B at 25 min and 12% B at 30 min. The column equilibration time was 8 min. External standards of 10 µM amino acids were injected after every 20 samples. Chromatograms were evaluated with HP Chem-Station software. Concentration of GABA measured in dialysate samples collected during drug applications were related to averaged GABA concentration of control samples collected for an hour prior to drug application and were given as percent changes. The basal GABA concentration of the dialysate samples was found to be 0.075±0.02 µM in the hippocampus of rats in accordance with data from other studies (0.069±0.013 µM) [53].

### [^3^H]GABA efflux from acute rat hippocampal slices

Radiolabeling of 400 µm thick acute slices was conducted in 200 µl of ACSF in a glass tube. This ACSF contained [^3^H]GABA (100 nM, Amersham) with 900 nM unlabeled GABA, and GABA transaminase (GABA-T) inhibitors vigabatrin (1 mM, Tocris) or gabaculine (250 µM, Tocris). To preferentially label the cytosolic over the vesicular pools, incubation of slices at 30°C lasted for 10 min [54]. Slices were washed with fresh ACSF containing GABA-T inhibitors, thereafter placed in a custom-built closed micro-volume (50 µl) chamber secured in a water bath thermostated at 30°C [55]. Thereafter, superfusion of ACSF (30°C) with 100 µM NNC-711 (Tocris), and vigabatrin (1 mM) or gabaculine (250 µM) at a rate of 0.5 ml/min was started. The superfusate was collected in 0.5 ml fractions. After washing for 10 min, 10 fractions were collected to define the basal release of radioactivity (‘pre-stimulus’). At this point, the hippocampal slice was exposed to 100 µM Glu for a period of 10 min (‘drug application’), followed by further 10 min of washing with ACSF (‘washout’). Radioactivity of each 0.5 ml fraction counted in 3 ml HiSafe 3 scintillation mixture was expressed as the percentage of the actual radioactivity content of the slice (fractional release). Fractional release data from individual slices were normalized to a first order exponential curve (obtained by fitting the pre-stimulus and the last 3 washout values). Normalized percent efflux data from fractions of ‘pre-stimulus’ (1–10 min) versus ‘drug application’ (11–20 min) periods were averaged and compared by statistical analysis.

### Double immunolabeling of GAT-1 and GAT-3 with neuronal and glial markers

Sections were immunolabeled first for GAT-1 or GAT-3 using affinity-purified polyclonal antisera (cat. #AB1570, lot #LV1436658 for GAT-1, and cat. #AB1574, lot #0512017869 for GAT-3, Chemicon, Temecula, CA). The epitope for the anti-GAT-1 antiserum was the C-terminus of rat GAT-1 (aa 588–599). The epitope for the anti-GAT-3 antiserum was the C-terminus of rat GAT-3 (aa 607–627) coupled to keyhole limpet hemocyanin. No cross-reactivity to the C-termini of other transmitter transporters was detected for either antiserum (see manufacturer's technical data sheet). The immunostaining was performed by using FITC-tyramide fluorescent amplification immunocytochemistry for GAT isoforms followed by Alexa594 labeling of the neuronal or glial markers. Briefly, free-floating brain sections were pretreated in phosphate buffer (pH = 7.4; PB) contaning 0.5% Triton X-100 and 3% bovine serum albumin for 1 hour. Then they were incubated with primary antibody against GAT-1 (1∶200) or GAT-3 (1∶150) in PB containing 0.5% Triton X-100, 3% bovine serum albumin, and 0.1% sodium azide for 48 hours at room temperature. Sections were then incubated in biotin-conjugated donkey anti-rabbit secondary antibody at 1∶500 (Jackson ImmunoResearch, West Grove, PA) for 2 hours, followed by incubation in avidinbiotin- horseradish peroxidase complex (ABC) at 1∶300 (Vectastain ABC Elite kit, Vector, Burlingame, CA) for 2 hours. Then sections were treated with fluorescein isothiocyanate (FITC)-tyramide (1∶8000) and 0.003% H_2_O_2_ in Tris-HCl buffer (0.05 M, pH 8.2) for 8 minutes. After washing, the sections were incubated overnight in either of the following mouse monoclonal primary antibodies: anti-glial fibrillary acidic protein, a marker of astrocytes (GFAP; 1∶250; cat. #sc-33673, Santa Cruz Biotechnology, Santa Cruz, CA), NeuN, a marker of neuronal cell bodies (1∶250; cat. #MAB377, lot #25070259, Chemicon), or anti-synaptophysin, a marker of presynaptic terminals (1∶100; cat. #M0776, CakoCytomation, Glostrup, Denmark). After incubation in the primary antibodies, sections were incubated in Alexa 594 donkey anti-mouse secondary antibody (1∶400; Molecular Probes) for 2 hours and mounted on positively charged slides (Superfrost Plus, Fisher Scientific, Fair Lawn, NJ), and coverslipped in antifade medium (Prolong Antifade Kit, Molecular Probes, Eugene, OR).

Sections were examined by using an Olympus BX60 light microscope also equipped with fluorescent epiillumination. Images were captured at 2,048×2,048 pixel resolution with a SPOT Xplorer digital CCD camera (Diagnostic Instruments, Sterling Heights, MI) by using 4–40X objectives. Confocal images were acquired at 1,024×1,024 pixel resolution with a Nikon Eclipse E800 confocal microscope equipped with a BioRad Radiance 2100 Laser Scanning System by using 20–40X objectives at optical thicknesses of 1–5 µm. Contrast and sharpness of the images were adjusted by using the levels and sharpness commands in Adobe Photoshop CS 8.0 (Adobe Systems, San Jose, CA). Full resolution was maintained until the photomicrographs were cropped and assembled for printing, at which point images were adjusted to a resolution of 300 dpi.

### NMR studies

NPMV samples for NMR experiments were prepared as described before [49]. NPMV fractions from the whole cortex were homogenized in 1 ml HEPES buffer containing 300 µM [^13^C]Glu and were incubated at 30°C for 30 min. The homogenate was then centrifuged at 3500 *g* for 15 min. The supernatant (extracellular fraction) was stored and the pellet (intracellular fraction) was suspended in 1 ml HEPES buffer and frozen (−80°C) and thawed 3 times. In the NMR experiments on brain slices, 13 hippocampal slices were put in a 2 ml centrifuge tube and the ACSF was replaced with 1 ml ACSF containing 300 µM [^13^C]Glu. The slices were incubated at 37°C for 30 min. Afterwards, the samples were frozen (−80°C) and thawed 3 times.

NMR experiments were carried out at 30°C on a *Varian NMR SYSTEM* (600 MHz for ^1^H) four-channel spectrometer using a 5 mm indirect detection triple resonance (^1^H^13^C^15^N) z-axis gradient probe. Samples (200 µl) were placed into 5 mm Shigemi NMR tubes. Gradient-enhanced ^1^H/^13^C heteronuclear correlation spectra (^13^C-HSQC) [56] were acquired with a ^1^H spectral width of 9600 Hz and 2048 complex points, zero-filled to a total of 4096. In the ^13^C dimension, with a spectral width of 21300 Hz, 96 hypercomplex increments were collected and linear predicted to a total of 384 points. Gaussian and exponential weighting functions were applied in the F2 dimension, while the indirectly detected dimensions were Gaussian weighted only. Spectra were processed in VnmrJ®. Proton chemical shifts were referenced to DSS at 0.00 ppm. The carbon dimension was referenced indirectly by taking into account the difference in the gyromagnetic ratios of ^13^C and ^1^H. NMR Spectroscopic Data for GABA: ^1^H NMR (599.9 MHz, DSS, 30°C): δ• 1.80 (H_β_), 2.19 (H_α_), 2.92 (H_γ_); ^13^C NMR (125.8 MHz, DSS, 30°C): δ• 23.9 (C_β_), 34.8 (C_α_), 39.7 (C_γ_).

### Semicarbazide treatment

In experiments where semicarbazide (SCB) was applied, animals were treated with SCB 3 hours before decapitation in 100 mg/kg dose intraperitoneally. During the experiments on NPMV fractions and acute hippocampal slices SCB was present in 2 mM concentration. Efficiency of SCB treatment was confirmed by the lack of [^13^C]GABA formation in rat cortical NPMV fraction (Figure 5A) and the reduced [^13^C]GABA formation in acute hippocampal slices (data not shown) following exposure of preparations from treated animals to [^13^C]Glu. Efficiency of SCB treatment was also justified by the clear signs of epileptic seizures induced in treated juvenile rats used for radiotracer release experiments.

### Statistics

Unless stated otherwise data are expressed as means±S.D. and were analyzed using paired *t*-test or one-way analysis of variances (ANOVA, OriginPro 7.5). A value of P<0.05 was considered significant.
